# Heart–Liver Interactions: Pathophysiology and Diagnosis of Hypoxic Hepatitis, Congestive Hepatopathy, and Cirrhotic Cardiomyopathy

**DOI:** 10.3390/diagnostics16193126

**Published:** 2026-09-26

**Authors:** Dusan Popovic, Nikola Blagojevic, Sanja Dragasevic Vucicevic, Milica Dragovic, Dragana Blagojevic, Tijana Glisic, Dragana Mijac, Milica Stojkovic-Lalosevic, Branka Filipovic, Jelena Bogdanovic, Ljiljana Bogdanovic, Natasa Nikolic, Marija Stojanovic

**Affiliations:** 1 Faculty of Medicine, University of Belgrade, 11000 Belgrade, Serbia; dragasevicsanja@gmail.com (S.D.V.); tijana.glisic78@gmail.com (T.G.); draganamijac@gmail.com (D.M.); drmilicastojkovic@gmail.com (M.S.-L.); branka.filipovic3@gmail.com (B.F.); jeca.bogdanovic@yahoo.com (J.B.); ljbogdanovic77@yahoo.com (L.B.); nanika1984@yahoo.com (N.N.); mrj.stojanovic@gmail.com (M.S.); 2 Clinic for Internal Medicine, Clinical Hospital Center “Dr Dragisa Misovic-Dedinje”, 11040 Belgrade, Serbia; n.blagojevic0205@gmail.com (N.B.); milicadragovic50@gmail.com (M.D.);; 3 Clinic for Gastroenterohepatology, University Clinical Centre of Serbia, 11000 Belgrade, Serbia; 4 Clinic for Endocrinology, Diabetes and Metabolic Diseases, University Clinical Centre of Serbia, 11000 Belgrade, Serbia; 5 Institute of Pathology, Faculty of Medicine, University of Belgrade, 11000 Belgrade, Serbia; 6 Clinic for Infectious and Tropical Diseases, University Clinical Centre of Serbia, 11000 Belgrade, Serbia; 7 Institute of Medical Physiology “Richard Burian”, Faculty of Medicine, University of Belgrade, 11000 Belgrade, Serbia

**Keywords:** heart–liver axis, congestive hepatopathy, hypoxic hepatitis, cirrhotic cardiomyopathy, heart failure, echocardiography, multimodal imaging, biomarkers, liver fibrosis, cardiohepatic syndrome

## Abstract

The heart and liver are closely interconnected through complex hemodynamic, neurohumoral, inflammatory, and metabolic mechanisms. Dysfunction of either organ may adversely affect the other, giving rise to a broad spectrum of cardiohepatic disorders with important diagnostic and prognostic implications. This narrative review is based on a literature search strategy of PubMed and the Web of Science Core Collection covering publications from January 2000 through June 2026, with a focus on hypoxic hepatitis, congestive hepatopathy, and cirrhotic cardiomyopathy. Cardiac diseases may induce liver injury through acute hypoperfusion or chronic venous congestion, resulting in distinct clinical entities characterized by different pathophysiological mechanisms, laboratory findings, imaging features, and therapeutic approaches. Conversely, advanced liver disease may be associated with impaired cardiovascular function, including cirrhotic cardiomyopathy, a condition that frequently remains clinically silent until physiological or therapeutic stress unmasks impaired cardiac reserve. Recent advances in multimodal imaging, including echocardiography with myocardial deformation analysis, abdominal ultrasonography with Doppler evaluation, elastography, computed tomography, and cardiac magnetic resonance, together with emerging biomarkers, have provided additional tools for the recognition and characterization of these disorders, although the clinical value of some newer techniques and biomarkers remains under investigation. Nevertheless, differentiating congestion-related liver injury from progressive fibrosis and identifying subclinical cardiac dysfunction remain important clinical challenges. A comprehensive understanding of the bidirectional interactions between the heart and liver is important for timely diagnosis, accurate risk stratification, and appropriate management. Future studies should focus on standardized diagnostic pathways, validation of novel imaging techniques and biomarkers, and multidisciplinary strategies that may improve outcomes in patients with complex cardiohepatic disease.

## 1. Introduction

The interactions between the heart and the liver represent a clinically and pathophysiologically important bidirectional relationship between these two organs. Although the terms hepatocardiac syndrome and cardiohepatic syndrome are used in the literature, no universally accepted definition of these entities has yet been established. The contemporary concept of the heart–liver axis describes the complex and dynamic bidirectional relationship between the heart and the liver [1].

Within this framework, heart–liver interactions can be considered from two principal perspectives: the impact of cardiac diseases on liver structure and function, and the impact of liver diseases on cardiac structure and function. In addition, several systemic disorders simultaneously affect both organs [2,3,4].

These interactions are of considerable clinical importance, particularly in patients with heart failure and other critical illnesses, in whom liver dysfunction may remain unrecognized or be misinterpreted, while also providing important prognostic information [5,6].

Contemporary diagnostic imaging modalities have further advanced the understanding of these disorders by enabling detailed assessment of hemodynamic alterations and structural changes in the liver in the setting of chronic venous congestion [1].

Although individual aspects of heart–liver interactions have been discussed in previous reviews, most have focused on specific diseases or on particular aspects of their pathophysiology and diagnosis [1,2,6,7]. In clinical practice, hypoxic hepatitis, congestive hepatopathy, and cirrhotic cardiomyopathy may occur in overlapping clinical settings, making their recognition and differentiation difficult. The increasing use of advanced imaging and new biomarkers has provided additional diagnostic information, but their clinical usefulness is not always clear. The aim of this review is to summarize the current knowledge of the bidirectional heart–liver relationship, with a focus on the pathophysiology, clinical presentation, and diagnosis of hypoxic hepatitis, congestive hepatopathy, and cirrhotic cardiomyopathy. We also discuss the main laboratory, imaging, and biomarker findings that may help distinguish these conditions and assess their clinical significance.

## 2. Materials and Methods

A predefined literature search strategy was performed in PubMed and the Web of Science Core Collection, covering publications from 1 January 2000 through 30 June 2026. The literature search strategy was further refined and documented to improve methodological transparency and reproducibility. The search strategy comprised four complementary domains addressing: (1) ischemic/hypoxic hepatitis; (2) congestive hepatopathy and related cardiac liver disease; (3) cirrhotic cardiomyopathy and cirrhosis-associated cardiac dysfunction; and (4) the heart–liver axis and cardiohepatic syndromes, with emphasis on diagnostic and pathophysiological aspects. Searches were restricted to English-language publications. Complete database-specific search strategies, including Boolean combinations, search fields, date limits, and retrieved records, are provided in Supplementary Table S1. The searches yielded 1040 records in PubMed and 1914 English-language records in Web of Science. These numbers represent retrievals from overlapping thematic searches and therefore do not correspond to the number of unique publications. As this was a narrative review, no formal PRISMA-based screening or deduplication process was undertaken.

Relevant original studies, systematic reviews, meta-analyses, clinical guidelines, and consensus statements published in English were considered. Publications were selected based on their relevance to the objectives of this review, with particular attention to recent advances in diagnostic imaging and biomarker-based assessment. Publications that were outside the predefined clinical domains or did not provide relevant diagnostic or pathophysiological information were not considered. Additional references were identified through manual screening of reference lists from selected articles.

Because this was a narrative review, study selection was based on relevance and clinical applicability rather than a formal systematic-review protocol. No formal risk-of-bias assessment or quantitative evidence grading was performed. Diagnostic thresholds and quantitative findings were interpreted in the context of the underlying study population, clinical setting, and diagnostic definitions, and original sources were preferentially consulted when available.

Literature selection and verification were performed by D.P., N.B., and M.S., and unclear issues were resolved by consensus. Particular attention was given to checking diagnostic thresholds, quantitative findings, and reference assignments against the original publications when available. The literature was considered qualitatively according to the type and relevance of the evidence, with particular emphasis on clinical guidelines, consensus statements, systematic reviews, meta-analyses, and original diagnostic studies, as appropriate to the specific clinical question.

This review is divided into two parts. The first part focuses on liver dysfunction resulting from cardiac disease, primarily hypoxic hepatitis and congestive hepatopathy, whereas the second part addresses the effects of liver disease on cardiac structure and function.

## 3. Hypoxic Hepatitis

### 3.1. Epidemiology of Hypoxic Hepatitis

Hypoxic hepatitis (HH), traditionally referred to as ischemic hepatitis (IH) or “shock liver,” is characterized by acute hepatocellular injury resulting from impaired hepatic oxygen delivery, most commonly in the setting of cardiac, circulatory, or respiratory failure [8].

In the literature, these terms are generally used to describe the same clinical syndrome, although there are some differences in the pathophysiological mechanisms they emphasize. The term hypoxic hepatitis is broader and better reflects the current understanding of this condition, as it emphasizes that the key mechanism is inadequate oxygen availability to hepatocytes, which may result from ischemia, venous congestion, systemic hypoxemia, or a combination of different factors [9,10]. Therefore, the presence of shock is not required for the development of hypoxic hepatitis [9,10]. The term ischemic hepatitis places greater emphasis on reduced hepatic blood flow and has historically been associated with hypoperfusion and shock, hence the use of the term “shock liver.” In accordance with current terminology, in this review we use the terms hypoxic hepatitis and ischemic hepatitis interchangeably, with hypoxic hepatitis being the preferred term.

Approximately 90% of cases occur in association with heart failure, respiratory failure, or septic shock [2,11]. The reported prevalence ranges from 1 to 1.6 per 1000 hospital admissions [11,12], with the highest incidence observed in intensive care units (1–11.9%) [11,12]. Ischemic hepatitis occurs in approximately 22% of patients with cardiogenic shock and in 13.8% of those with septic shock [11,13]. The condition predominantly affects critically ill patients and is associated with substantial mortality [14]. In a large systematic review, a preceding acute cardiovascular event was identified in approximately 78% of patients with ischemic hepatitis, while sepsis was present in approximately 23% and documented hypotension in approximately 53% of cases [12].

Liver dysfunction in patients with cardiogenic shock is not merely a marker of systemic hypoperfusion but also carries important prognostic significance, as the development of hypoxic hepatitis is associated with more than a twofold increase in the risk of in-hospital mortality [15].

### 3.2. Liver Physiology (Key Considerations) and Pathophysiology of Hypoxic Hepatitis

A thorough understanding of normal liver physiology is essential for understanding hypoxic hepatitis and congestive hepatopathy.

The liver is a highly perfused organ, receiving approximately 20–25% of the cardiac output [16]. Several physiological mechanisms protect the liver from ischemic injury. The first protective mechanism is its dual blood supply. The liver receives blood from both the portal vein and the hepatic artery. The portal vein delivers oxygen-poor blood from the abdominal organs and accounts for approximately 70–80% of total hepatic blood flow, whereas the hepatic artery provides the remaining 20–30% of oxygen-rich arterial blood [6,16]. A reduction in portal venous flow is partially compensated by an increase in hepatic arterial flow through the hepatic arterial buffer response [2,6]. This mechanism is mediated by adenosine washout. Adenosine is continuously released around the terminal branches of the hepatic artery and normally cleared by portal venous blood flow. When portal flow decreases, adenosine accumulates, resulting in arteriolar vasodilation and increased hepatic arterial perfusion [2]. However, this compensatory mechanism has limited capacity, and once exceeded, tissue hypoxia develops.

Another important protective mechanism is the unique structure of the hepatic sinusoids. These vascular channels are lined by specialized fenestrated endothelial cells and lack a continuous basement membrane, allowing high permeability and efficient oxygen diffusion. In addition, hepatocytes possess a considerable capacity for oxygen extraction, providing further protection against transient reductions in hepatic perfusion [3,11].

The hepatic acinus is the functional unit of the liver and is divided into three zones according to the distance from the terminal branches of the portal vein and hepatic artery. Zone 1 (the periportal zone) is located closest to the incoming blood supply and receives blood with the highest oxygen and nutrient content. Zone 2 represents the intermediate region, whereas Zone 3 (the centrilobular zone) is farthest from the arterial blood supply and physiologically has the lowest oxygen concentration. Consequently, hepatocytes in Zone 3 are the most susceptible to hypoxia and ischemic injury, explaining the predominance of centrilobular necrosis in both ischemic hepatitis and congestive hepatopathy [2].

Hypoxic hepatitis is characterized by diffuse liver injury resulting from hepatic hypoperfusion and most commonly develops in the setting of systemic hypotension superimposed on pre-existing cardiac dysfunction [14].

Systemic oxygen delivery (DO_2_) depends directly on cardiac output (CO), hemoglobin concentration (Hb), and arterial oxygenation (SaO_2_) and can be calculated using the following equation:

DO_2_ = CO × [(1.34 × Hb × SaO_2_) + (0.003 × PaO_2_)] × 10

Acute conditions such as acute myocardial infarction, severe arrhythmias, or cardiac tamponade may result in an abrupt reduction in cardiac output, systemic perfusion, and oxygen delivery, thereby predisposing patients to ischemic hepatitis. Pre-existing hepatic venous congestion is believed to markedly increase hepatocyte susceptibility to reduced oxygen delivery and thereby contribute to liver injury [17]. In cardiogenic shock, liver injury results from the combined effects of impaired systemic perfusion and passive hepatic congestion, further illustrating the complex mechanisms underlying the cardio-hepatic syndrome [15]. In respiratory failure, the predominant mechanism of hepatic ischemia is reduced arterial oxygen saturation. In septic shock, oxygen demand is increased while hepatocytes are unable to utilize oxygen efficiently, a phenomenon referred to as dysoxia. Endotoxins and inflammatory mediators released during septic shock also play a significant role in the pathogenesis of ischemic hepatitis [8,11].

Although ischemia is the initiating event in ischemic hepatitis, liver injury continues after restoration of blood flow through a process known as ischemia–reperfusion injury [18]. This process is analogous to reperfusion injury observed following acute myocardial infarction. The underlying mechanisms include oxidative stress, Kupffer cell activation, polymorphonuclear leukocyte activation, and disturbances of the hepatic microcirculation [18]. Increased generation of reactive oxygen species represents one of the principal mechanisms of hepatocellular injury, leading to lipid peroxidation of cell membranes, mitochondrial dysfunction, and progressive loss of hepatocyte viability [19,20].

### 3.3. Clinical Presentation of Hypoxic Hepatitis

The clinical presentation of hypoxic hepatitis is usually dominated by the manifestations of the underlying condition responsible for hepatic injury, including heart failure, respiratory failure, septic shock, or other critical illnesses. Liver-related symptoms are generally nonspecific and include nausea, vomiting, dull abdominal pain, and, at later stages, jaundice, oliguria, and asterixis [8]. Patients may also develop altered mental status manifested by confusion and agitation. Impaired consciousness in these patients is usually attributable to cerebral hypoxia and hemodynamic instability rather than liver failure or hepatic encephalopathy [8]. Some patients remain entirely asymptomatic. Abdominal symptoms may develop after a latent period of 2–24 h following the acute precipitating event [3,8,11,21].

### 3.4. Diagnosis of Hypoxic Hepatitis

In addition to medical history and physical examination, laboratory testing plays a central role in the diagnosis of hypoxic hepatitis. Hypoxic hepatitis is one of the most common causes of marked elevation of serum aminotransferases. Studies have shown that approximately 40% of hospitalizations associated with severe aminotransferase elevation can be attributed to ischemic hepatitis [22]. Marked elevations of aspartate aminotransferase (AST) and alanine aminotransferase (ALT), typically exceeding 20 times and sometimes reaching much higher levels, are characteristic of hypoxic hepatitis. In some patients, aminotransferase levels may reach several hundred times the upper reference limit [8,17,22]. In most cases, the AST/ALT ratio exceeds 1, reflecting the predominant injury to Zone 3 of the hepatic acinus, where AST activity is greatest [22]. The degree of aminotransferase elevation is not diagnostic by itself and should be interpreted in relation to the clinical setting and timeline pattern of enzyme changes [8,22,23]. Similar elevations in aminotransferases may also occur in drug-induced liver injury, autoimmune hepatitis, acute viral hepatitis, and other conditions, all of which should be considered in the differential diagnosis [3,5,8,23]. The principal causes of marked aminotransferase elevation relevant to the differential diagnosis of ischemic hepatitis are summarized in Table 1.

In addition to marked aminotransferase elevation, hypoxic hepatitis is characterized by a substantial increase in lactate dehydrogenase (LDH), typically reaching 10–20 times the upper limit of normal. An ALT/LDH ratio < 1.5 is considered characteristic of ischemic hepatitis [8]. This ratio should be regarded as a supportive rather than definitive diagnostic criterion, and its interpretation depends on the laboratory reference ranges and clinical context. Although hyperbilirubinemia is common, it is generally mild, whereas alkaline phosphatase levels are usually normal or only minimally elevated [22].

A characteristic feature of hypoxic hepatitis is a decline in ALT levels of more than 50% within 72 h, which represents an important diagnostic clue [8,23]. Such a rapid decline is generally not observed in drug-induced liver injury, viral hepatitis, or alcoholic hepatitis [8].

In addition to the characteristic biochemical pattern dominated by elevated AST, ALT, and LDH, the temporal profile of liver enzyme abnormalities is also highly characteristic. Serum AST, ALT, and LDH levels typically peak within 1–3 days after the acute ischemic event, and, provided that the underlying cause is successfully treated, usually return to normal within 7–10 days [8].

The diagnosis of hypoxic hepatitis requires the presence of cardiac, circulatory, or respiratory failure; a transient increase in aminotransferase levels (≥20 times the upper limit of the reference range); and exclusion of other causes of liver injury [10].

## 4. Congestive Hepatopathy

Congestive hepatopathy most commonly develops in patients with chronic right-sided heart failure or other conditions associated with persistently elevated right-sided cardiac pressures. It is mainly the result of chronic hepatic venous congestion and differs from hypoxic hepatitis, which is an acute form of liver injury related to reduced oxygen delivery. The two conditions may occur together, particularly in patients with severe cardiac dysfunction, where chronic congestion can make the liver more vulnerable to acute hypoxic injury.

### 4.1. Epidemiology of Congestive Hepatopathy

The prevalence increases with the duration and severity of heart failure and is therefore most frequently observed in patients with advanced cardiac disease and in candidates for heart transplantation. The reported frequency varies depending on what is considered evidence of liver involvement. Abnormal liver tests, signs of hepatic venous congestion on imaging, clinically evident congestive hepatopathy, and advanced fibrosis or cardiac cirrhosis do not represent the same degree of liver disease. In the study by Balcar et al., hepatic congestion was present in 54% of patients on the heart transplant waiting list, while congestive hepatopathy was reported in 29% [13]. Following the Fontan procedure, the vast majority of patients develop some degree of congestive hepatopathy, whereas clinically significant Fontan-associated liver disease (FALD) may develop in a substantial proportion of patients [24,25].

### 4.2. Pathophysiology of Congestive Hepatopathy

Conditions such as heart failure, congenital heart disease, mitral stenosis, severe tricuspid regurgitation, and constrictive pericarditis lead to elevated right atrial pressure, which is transmitted retrogradely to the inferior vena cava and subsequently to the hepatic and central veins. Persistent congestion impairs sinusoidal blood flow and, particularly in patients with reduced cardiac output, contributes to centrilobular hypoxia and hepatocellular injury [26,27]. Unlike the abrupt hemodynamic insult characteristic of hypoxic hepatitis, the injury in congestive hepatopathy is usually chronic and progressive. Over time, fibrosis may develop, and a subset of patients progresses to advanced chronic liver disease with clinically significant hepatic dysfunction. Hepatic injury activates hepatic stellate cells, promoting their transformation into myofibroblasts and initiating collagen deposition and fibrogenesis [26]. Several cytokines, including tumor necrosis factor-α (TNF-α), interleukin-6 (IL-6), and transforming growth factor-β (TGF-β), also contribute to hepatic injury and fibrosis [26].

Recent studies indicate that sinusoidal thrombosis and mechanical forces generated by chronically elevated venous pressure play an important role in the development of fibrosis. These observations have led to the concept of the “congestive escalator,” according to which chronic venous congestion progressively promotes parenchymal loss and fibrosis even in the absence of pronounced inflammation [2]. Progressive fibrosis may ultimately result in cardiac cirrhosis, which shares many pathological and clinical features with cirrhosis of other etiologies. An important characteristic of cardiac cirrhosis is the development of portal hypertension, which underlies many of its complications. As with other forms of cirrhosis, cardiac cirrhosis may also predispose to the development of hepatocellular carcinoma.

### 4.3. Clinical Presentation of Congestive Hepatopathy

The clinical presentation of congestive hepatopathy is predominantly characterized by symptoms and signs of heart failure resulting from structural or functional cardiac abnormalities leading to systolic and/or diastolic dysfunction [8]. Typical manifestations include dyspnea, orthopnea, paroxysmal nocturnal dyspnea, and peripheral edema [8]. Fatigue and generalized weakness are common features of advanced congestive hepatopathy and reflect the combined effects of chronic heart failure and impaired liver function. Some patients may experience nausea and vomiting secondary to impaired bile flow and maldigestion [5].

Abdominal manifestations are also common. Patients frequently report abdominal discomfort and a dull pain in the right upper quadrant, which results from hepatic congestion and stretching of Glisson’s capsule. Hepatomegaly is a common physical finding, and a positive hepatojugular reflux may be detected on examination. As the disease progresses to cardiac cirrhosis, patients may develop the full spectrum of complications associated with cirrhosis irrespective of etiology. Advanced cardiac cirrhosis may therefore be complicated by portal hypertension, ascites, splenomegaly, hypersplenism, gastroesophageal varices, and hepatic encephalopathy. Although these complications occur less frequently than in cirrhosis of other etiologies, their presence indicates advanced liver disease and carries important prognostic implications.

### 4.4. Diagnosis of Congestive Hepatopathy

The diagnosis of congestive hepatopathy is based on a comprehensive clinical evaluation, including a detailed medical history and physical examination, complemented by laboratory testing and imaging studies, while liver biopsy is required only in exceptional cases. Diagnosis therefore depends largely on a high index of clinical suspicion.

Patients with congestive hepatopathy typically demonstrate mild elevations of serum aminotransferases, considerably less pronounced than those observed in hypoxic hepatitis. Serum AST and ALT levels are generally elevated to approximately two- to threefold the upper limit of normal, but the degree of abnormality varies with the severity and duration of congestion and the underlying cardiac condition [8]. Mild cholestatic abnormalities usually predominate, including elevations in bilirubin, alkaline phosphatase (ALP), and gamma-glutamyl transferase (GGT) [8]. Differential diagnosis of hypoxic hepatitis and congestive hepatopathy is shown in Table 2. Conventional liver biochemical tests have limited value for assessing the degree of fibrosis in congestive hepatopathy, as they often remain normal or only mildly abnormal despite advanced histopathological changes [28]. Prolonged prothrombin time and hypoalbuminemia may occur as a consequence of hepatic dysfunction, inflammation, hemodilution, and malnutrition [8].

When ascites is present, diagnostic paracentesis plays an important role in excluding alternative etiologies. In cardiac ascites, the serum–ascites albumin gradient (SAAG) is typically >1.1 g/dL, ascitic fluid protein concentration is ≥2.5 g/dL, and both lactate dehydrogenase (LDH) levels and erythrocyte counts are elevated [8]. In a prospective diagnostic accuracy study of patients with new-onset ascites, serum BNP > 364 pg/mL differentiated heart-failure-related ascites from other causes with a sensitivity of 98% and specificity of 99% [29]. Measurement of N-terminal pro-B-type natriuretic peptide (NT-proBNP) in serum and ascitic fluid may further support the identification of heart failure as the underlying cause of ascites [3,5,8]. In a small study including 58 patients with known cirrhosis and 18 patients diagnosed with heart failure, NT-proBNP levels were measured in both serum and ascites. The authors reported that a serum NT-proBNP cutoff of 1000 pg/mL showed 100% sensitivity for ruling out cirrhosis as the cause of ascites [30].

Imaging plays a central role in the diagnosis of congestive hepatopathy. Echocardiography identifies heart failure and structural cardiac abnormalities responsible for hepatic congestion. Abdominal ultrasonography may demonstrate hepatomegaly, dilated hepatic veins (>8–10 mm), and ascites [6]. Gallbladder wall thickening may also be observed as a consequence of ascites and hypoalbuminemia. In addition, ultrasonography may reveal morphological liver changes, including coarse parenchymal echotexture, a nodular liver surface, widened fissures, and hypertrophy of the caudate lobe [6]. These findings do not necessarily indicate cirrhosis, as they may also reflect nodular regenerative hyperplasia secondary to chronic hepatic congestion [6].

In addition to conventional B-mode ultrasonography, pulsed-wave (PW) Doppler provides valuable hemodynamic information. Under normal conditions, hepatic venous flow demonstrates a triphasic waveform reflecting pressure changes throughout the cardiac cycle. In congestive hepatopathy, this pattern progressively changes to biphasic or monophasic waveforms [31]. Doppler examination may also demonstrate increased pulsatility, phasic flow, and occasionally reduced portal venous flow velocity. Compensatory increases in hepatic arterial blood flow may likewise be detected using PW Doppler.

It is important to be aware of the limitations of ultrasound and Doppler assessment. Ultrasonography can indicate hepatic venous congestion and findings suggestive of portal hypertension, but it cannot reliably establish advanced liver fibrosis or cirrhosis. Hepatic venous dilatation and Doppler flow patterns mainly reflect hemodynamic changes and venous congestion and should not be used as the sole diagnostic criteria for fibrosis.

Caution is also needed when interpreting ultrasound parameters suggestive of portal hypertension, such as portal vein diameter, portal vein flow velocity, and flow phasicity. These parameters may be affected by cardiac function, heart rhythm, respiration, intravascular volume, recent food intake, as well as the experience of the operator, patient position, and acquisition technique. These parameters should be interpreted in the context of the patient’s clinical and hemodynamic status.

In recent years, elastographic techniques have assumed an increasingly important role. However, in patients with congestive hepatopathy, increased liver stiffness does not necessarily reflect the severity of fibrosis, as it may simply result from hepatic venous congestion [32]. Elastography findings should therefore always be interpreted in the context of the patient’s clinical presentation and hemodynamic status [2,6]. In patients with Fontan-associated liver disease, transient elastography has been associated with the presence and severity of FALD, including definitions of advanced FALD based on clinical and imaging features of portal hypertension. However, these findings do not establish reliable fibrosis staging, and liver stiffness may also be increased by Fontan-related venous congestion [33].

Computed tomography (CT) and magnetic resonance imaging (MRI) are key imaging modalities for evaluating congestive hepatopathy, as they allow comprehensive assessment of structural liver abnormalities, portal hypertension, and chronic hepatic venous congestion. They are particularly valuable for characterizing focal hepatic lesions, including benign regenerative nodules (focal nodular hyperplasia-like nodules) and early hepatocellular lesions. Because long-standing hepatic congestion may give rise to dysplastic nodules and hepatocellular carcinoma (HCC), imaging findings should always be interpreted in conjunction with the clinical context [6].

Liver biopsy is only rarely required for the diagnosis of congestive hepatopathy. It is primarily reserved for cases in which the diagnosis cannot be established using non-invasive methods or when histological findings may influence the decision regarding heart transplantation or combined heart–liver transplantation.

Macroscopically, the liver may exhibit the characteristic appearance of a nutmeg liver [2]. This appearance results from alternating dark red centrilobular areas of congestion and hemorrhage interspersed with paler periportal regions of preserved or steatotic hepatic parenchyma [2].

The characteristic histopathological findings of congestive hepatopathy include sinusoidal dilatation and congestion, predominantly involving acinar Zone 3, centrilobular hepatocyte atrophy, erythrocyte extravasation, and perisinusoidal fibrosis [2]. As the disease progresses, fibrous bridges may develop between adjacent central veins, producing the characteristic pattern of reversed lobulation, in contrast to most other chronic liver diseases in which fibrosis originates from the portal tracts [2,34].

### 4.5. Treatment of Hypoxic Hepatitis and Congestive Hepatopathy

There is no disease-specific treatment for hypoxic hepatitis or congestive hepatopathy. Management is primarily directed toward treating the underlying condition responsible for the hepatic injury. Therapy for hypoxic hepatitis is largely supportive and focuses on correcting hemodynamic disturbances and reversing the underlying cause of hepatic hypoperfusion. In recent years, the potential role of N-acetylcysteine (NAC) has been investigated; however, current evidence is insufficient to support its routine use [14,35].

Since cardiovascular disorders are a major cause of morbidity in these patients, treatment of the underlying cardiac disease should be guided by current heart failure recommendations. In patients with heart failure with reduced ejection fraction (HFrEF), treatment generally includes angiotensin receptor–neprilysin inhibitors (ARNIs), angiotensin-converting enzyme (ACE) inhibitors or angiotensin receptor blockers (ARBs), β-blockers, mineralocorticoid receptor antagonists (MRAs), and sodium–glucose cotransporter 2 (SGLT2) inhibitors [36,37]. In addition to their significant cardiorenal protective effects, SGLT2 inhibitors may also have a role in the treatment of ascites [38]. Their beneficial effects include a reduction in ascites, a decreased need for paracentesis, improvement in transaminase levels, and a reduced risk of hepatic decompensation and hepatocellular carcinoma [38,39,40]. Their use requires careful patient selection because of potentially serious adverse effects [38]. Heart transplantation remains the definitive treatment for selected patients with end-stage heart failure [41].

The principal therapeutic goal in congestive hepatopathy is the optimal treatment of heart failure. However, the benefits of this approach become limited once advanced hepatic fibrosis or cardiac cirrhosis has developed. The management of cardiac cirrhosis does not differ substantially from that of cirrhosis of other etiologies. Because ascites is one of the most common manifestations of decompensated cirrhosis, treatment includes diuretic therapy, which also provides benefit in patients with concomitant heart failure. Therapeutic paracentesis and albumin replacement are frequently required.

## 5. Cirrhotic Cardiomyopathy

### 5.1. Definition, Clinical Concept and Epidemiology of Cirrhotic Cardiomyopathy

Cirrhotic cardiomyopathy (CCM) is a form of cardiac dysfunction occurring in patients with cirrhosis in the absence of primary cardiac disease. It has traditionally been characterized by impaired cardiac contractile responsiveness to stress, altered diastolic relaxation, and electrophysiological abnormalities developing within the hyperdynamic circulation of cirrhosis [42]. However, current understanding has evolved from a disorder primarily attributed to hyperdynamic circulation toward a complex cardiohepatic syndrome involving myocardial, vascular, autonomic, inflammatory, and endothelial dysfunction [7,42].

Although patients with cirrhosis typically exhibit increased cardiac output and reduced systemic vascular resistance at rest, they often have impaired cardiovascular reserve and an inadequate response to physiological or pathological stress [42,43]. Consequently, cardiac dysfunction frequently remains clinically silent until triggered by conditions such as infections, gastrointestinal bleeding, transjugular intrahepatic portosystemic shunt (TIPS) placement, major surgery, or liver transplantation [7,44].

Historically, CCM diagnosis relied on conventional echocardiography, with preserved left ventricular ejection fraction (LVEF) and diastolic dysfunction considered its principal features. However, LVEF is an insensitive marker in cirrhosis because chronic vasodilation and reduced afterload may mask intrinsic myocardial dysfunction [7,42]. These limitations have shifted attention toward more sensitive diagnostic tools, including myocardial deformation imaging, stress echocardiography, circulating biomarkers, and comprehensive right ventricular assessment [7,45,46].

Recent international consensus criteria have further refined the diagnostic framework by incorporating contemporary cardiovascular imaging methodologies [42]. Global longitudinal strain (GLS) has emerged as a sensitive marker of subclinical systolic dysfunction, while increasing evidence supports the clinical relevance of right ventricular dysfunction, impaired ventriculoarterial coupling and chronotropic incompetence [45,46,47]. In parallel, biomarkers reflecting myocardial injury, wall stress, inflammation, endothelial dysfunction and extracellular matrix remodeling have gained importance as complementary tools for identifying patients at increased cardiovascular risk [7,44].

Contemporary studies consistently demonstrate that subclinical cardiovascular abnormalities are common in advanced cirrhosis when assessed using modern imaging techniques [42]. Therefore, current evaluation of CCM requires an integrated multimodal approach combining advanced echocardiography, myocardial deformation imaging, right ventricular assessment, circulating biomarkers and functional reserve testing to facilitate earlier diagnosis and improve cardiovascular risk stratification [45,46,48,49].

Reported prevalence of cirrhotic cardiomyopathy varies widely across published studies, largely reflecting differences in diagnostic criteria, patient populations, cirrhosis severity, and imaging methodologies. Earlier studies based predominantly on conventional echocardiographic parameters reported prevalence ranging from approximately 20% to 50%, whereas more recent investigations incorporating myocardial deformation imaging and contemporary diagnostic criteria have identified subclinical cardiac abnormalities in more than half of patients with advanced cirrhosis [7,42,45]. The absence of universally applied diagnostic standards continues to limit direct comparison between studies and likely contributes to underrecognition of the disease.

### 5.2. Pathophysiology of Cirrhotic Cardiomyopathy

CCM results from a complex interplay between systemic hemodynamic alterations, autonomic dysregulation, endothelial dysfunction, inflammation and myocardial remodeling [7,42]. Rather than an isolated myocardial disorder, CCM develops within the broader context of chronic liver disease and portal hypertension, where persistent hemodynamic stress, neurohumoral activation and inflammatory signaling progressively impair myocardial function and cardiovascular reserve [7,50].

One of the hallmarks of cirrhosis is the hyperdynamic circulatory state, characterized by reduced systemic vascular resistance, increased cardiac output and arterial vasodilation [7,50,51]. Splanchnic vasodilation, largely driven by excessive nitric oxide (NO) production and endothelial dysfunction, causes effective arterial hypovolemia despite expanded blood volume [50,52,53]. This activates the renin–angiotensin–aldosterone system (RAAS), sympathetic nervous system and vasopressin release, promoting sodium and water retention, chronic volume overload, myocardial remodeling and ultimately reduced cardiovascular reserve [7,50,52,54].

Despite elevated resting cardiac output, patients with cirrhosis often exhibit blunted cardiac responses to stress [42,43]. This is partly explained by autonomic dysfunction, impaired β-adrenergic signaling, reduced receptor density and abnormalities in intracellular calcium handling [51,54]. Together, these alterations limit both inotropic and chronotropic reserve, compromising the cardiovascular response to infection, hemorrhage, exercise, TIPS placement, or liver transplantation [43,44]. Evidence from animal models suggests that autonomic dysfunction is, at least in part, associated with decreased β-adrenergic receptor density and impaired cardiac responsiveness [55].

Inflammation and endothelial dysfunction further contribute to CCM. Chronic liver disease is associated with systemic inflammation, endotoxemia, oxidative stress and immune activation [7,52,53,54]. Increased production of nitric oxide, carbon monoxide, endocannabinoids and inflammatory mediators exerts negative inotropic effects while aggravating peripheral vasodilation and microvascular dysfunction [50,51]. Oxidative stress and mitochondrial dysfunction may additionally impair myocardial energetics within the cardiohepatic axis [7,47].

The inflammatory response observed in cirrhotic cardiomyopathy is largely driven by bacterial translocation and the subsequent entry of inflammatory triggers, particularly lipopolysaccharide (LPS), into the systemic circulation. Circulating LPS activates pattern-recognition receptors, such as Toll-like receptor 4 (TLR4), expressed on macrophages and other cell types [56]. This activation induces the production of pro-inflammatory cytokines, including tumor necrosis factor-α (TNF-α), interleukin-1β (IL-1β), and interleukin-6 (IL-6).

TNF-α has been shown to interfere with intracellular calcium signaling, thereby contributing to impaired cardiac function. Studies using a thioacetamide-induced rat model of cirrhosis demonstrated that inflammation significantly reduces phospholamban phosphorylation in the cardiomyocytes, leading to both systolic and diastolic dysfunction in this experimental model [57].

Taken together, these mechanisms support the concept of CCM as a systemic cardiohepatic disorder characterized by impaired cardiovascular reserve rather than isolated resting ventricular dysfunction.

### 5.3. Clinical Presentation of Cirrhotic Cardiomyopathy

Cirrhotic cardiomyopathy is frequently clinically silent at rest because the hyperdynamic circulatory state partially compensates for impaired myocardial reserve [7,42]. Many patients, consequently, remain asymptomatic until they are exposed to stress [7,42,50]. During stress, CCM reveals its symptoms: exertional dyspnoea, exercise intolerance, pulmonary edema, hypotension or overt heart failure. These observations emphasize that CCM represents a largely subclinical but clinically important cardiovascular complication of cirrhosis, underscoring the need for systematic cardiac assessment in patients with advanced liver disease.

### 5.4. Diagnosis of Cirrhotic Cardiomyopathy

Modern CCM assessment requires a multimodal imaging approach integrating electrocardiography, advanced echocardiographic imaging, functional testing and, in selected patients, cardiac magnetic resonance (CMR) [7,42]. Importantly, normal resting cardiac function does not exclude clinically relevant myocardial impairment, particularly in patients undergoing major hemodynamic stress [7,44]. An important limitation of the current diagnostic approach is the lack of specificity of individual cardiac findings for CCM. Abnormal GLS, diastolic indices, elevated cardiac biomarkers, reduced exercise capacity, and right-heart abnormalities may also occur in patients with cardiometabolic disease, alcohol-related cardiomyopathy, valvular disease, arrhythmias, renal dysfunction, anemia, infection or sepsis, abnormal volume status, and pulmonary disease. Reduced exercise capacity may additionally reflect sarcopenia and frailty. These conditions should therefore be considered before attributing an abnormal cardiac finding to CCM, particularly in patients with multiple comorbidities. The underlying etiology of liver disease should also be considered when interpreting cardiac abnormalities. Alcohol-associated liver disease may coexist with alcohol-related myocardial injury, while MASLD/MASH is frequently accompanied by obesity, diabetes, hypertension, and other cardiometabolic risk factors. Viral, autoimmune, and cholestatic liver diseases may have different systemic and cardiovascular manifestations, and iron-overload disorders may cause primary myocardial injury. Fontan-associated liver disease represents a distinct setting in which chronic venous hypertension and altered cardiac loading conditions may produce cardiac and hepatic abnormalities that cannot be interpreted using the same framework as conventional cirrhosis. Thus, the etiology of liver disease and the presence of concomitant cardiovascular disease should be considered when interpreting findings suggestive of CCM.

Most abnormal electrocardiographic (ECG) findings in cirrhosis are nonspecific. Prolongation of the corrected QT interval (QTc) (commonly defined as >440 ms in men and >460 ms in women) is the most frequently reported abnormality and has been associated with autonomic dysfunction, altered membrane electrophysiology, and severity of liver disease [44,58]. Additional ECG abnormalities include chronotropic incompetence, impaired heart rate variability and nonspecific ST-T wave changes.

Transthoracic echocardiography remains the cornerstone of CCM assessment and is currently incorporated into all major diagnostic frameworks. Standard evaluation includes left ventricular ejection fraction (LVEF), chamber dimensions, transmitral Doppler flow, tissue Doppler imaging, left atrial volume index (LAVI), and estimation of pulmonary pressures [42,44,59].

However, interpretation of conventional echocardiographic findings in cirrhosis may be complicated by hyperdynamic circulation and marked preload dependence. Reduced systemic vascular resistance may preserve or even increase LVEF despite impaired myocardial contractility, limiting the sensitivity of conventional systolic parameters for early disease detection [7,42,45]. According to the revised Cirrhotic Cardiomyopathy Consortium criteria, overt LV systolic dysfunction is defined by LVEF ≤ 50%.

Diastolic dysfunction remains one of the principal echocardiographic abnormalities in CCM. According to the Cirrhotic Cardiomyopathy Consortium criteria, advanced diastolic dysfunction is defined by the presence of at least three of four echocardiographic abnormalities: septal e′ velocity (<7 cm/s), E/e′ ratio ≥ 15, LAVI > 34 mL/m^2^ and peak tricuspid regurgitation velocity > 2.8 m/s. The interpretation of peak tricuspid regurgitation velocity requires caution in patients with pulmonary or portopulmonary hypertension, in whom elevated values may reflect pulmonary vascular disease rather than CCM. Nevertheless, interpretation of these parameters in cirrhosis may be challenging because ascites, sodium retention, tachycardia and dynamic intravascular volume changes substantially influence Doppler-derived measurements [7,42,47]. Right ventricular parameters are increasingly recognized as clinically relevant in cirrhosis, although they are currently considered complementary measures rather than independent diagnostic criteria for CCM. Echocardiographic Parameters for the Assessment of Cirrhotic Cardiomyopathy are shown in Table 3.

Speckle-tracking echocardiography (STE) has emerged as one of the most promising imaging modalities for detection of subclinical myocardial dysfunction in cirrhosis. GLS is considered more sensitive than conventional LVEF for identifying early systolic impairment [46]. The incorporation of GLS into the revised CCM criteria represents an important shift toward myocardial mechanics-based assessment. According to the contemporary criteria, LV systolic dysfunction may also be defined by absolute GLS < 18% [42]. Several studies have demonstrated impaired GLS values despite preserved LVEF, supporting the concept that latent systolic dysfunction may remain undetected using conventional echocardiographic assessment alone [46,49]. From a clinical perspective, impaired GLS has been associated with more advanced liver disease, reduced functional reserve, and adverse posttransplant cardiovascular outcomes in some cohorts [46,48]. However, interpretation of strain imaging in cirrhosis requires caution because hyperdynamic circulation and loading conditions may influence strain values, particularly in advanced disease stages.

A defining characteristic of CCM is impaired cardiovascular reserve during physiological or pharmacological stress testing despite apparently preserved resting cardiac function. Stress echocardiography may therefore reveal latent myocardial dysfunction not detectable under baseline conditions. Blunted contractile response and impaired chronotropic adaptation during exercise or dobutamine stress have been described in cirrhotic patients [7,59].

Stress-based assessment may be particularly important before TIPS implantation and liver transplantation, where abrupt hemodynamic changes can precipitate overt heart failure in patients with previously unrecognized CCM [7,44]. Accordingly, evaluation of functional reserve is recognized as a relevant component of comprehensive cardiovascular assessment in advanced cirrhosis.

Cardiopulmonary exercise testing (CPET) enables simultaneous evaluation of cardiovascular, pulmonary, and peripheral responses to exercise and may identify exercise intolerance not apparent during resting evaluation [60]. Reduced peak oxygen consumption (peak VO_2_), impaired exercise tolerance and abnormal ventilatory response patterns have all been described in cirrhotic populations. Lower peak VO_2_ values have been associated with impaired functional status and poorer pretransplant outcomes, suggesting potential prognostic value of CPET in advanced liver disease [60]. However, exercise intolerance in cirrhosis is multifactorial and may additionally reflect sarcopenia, frailty, anemia, pulmonary abnormalities and peripheral circulatory dysfunction rather than isolated myocardial impairment alone.

Compared with echocardiography, cardiac magnetic resonance (CMR) enables more reproducible assessment of ventricular volumes and myocardial tissue composition, including diffuse fibrosis, myocardial edema and extracellular matrix remodeling through T1 mapping and extracellular volume (ECV) analysis [7,47]. In addition, cost, limited availability and technical complexity currently restrict broader implementation of CMR in routine cardiovascular assessment of cirrhotic patients.

Biomarkers provide complementary information in the evaluation of CCM, particularly in patients with subclinical disease, although none has sufficient diagnostic specificity to establish the diagnosis independently, underscoring the need for an integrated multimodal approach.

Natriuretic peptides (BNP and NT-proBNP) are the most extensively studied biomarkers in cirrhotic cardiomyopathy and are associated with myocardial wall stress, cardiac remodeling, diastolic dysfunction, liver disease severity and echocardiographic markers of cardiac dysfunction [7,47,61,62,63]. Elevated BNP or NT-proBNP levels are not specific for cirrhotic cardiomyopathy, as they may reflect hyperdynamic circulation, volume overload, renal dysfunction, and advanced liver disease [44,63].

In the study by Saner et al., involving 157 liver transplant recipients with cirrhosis, BNP > 391 pg/mL was associated with echocardiographic diastolic dysfunction and higher mortality [62]. This threshold was reported in a transplant population and should not be considered a universal diagnostic cut-off for CCM. Cardiac troponins may be elevated in advanced cirrhosis, possibly reflecting myocardial stress or subclinical myocardial injury. Higher troponin concentrations have been associated with adverse outcomes in cirrhosis, but troponin elevation is not specific for CCM and should be interpreted in the clinical context [47,51].

In the study by Grzebyk et al., involving 87 patients with HCV-related cirrhosis, NT-proBNP showed the highest discriminatory value for CCM (AUROC 0.796), followed by hs-TnT (AUROC 0.753) [64]. Amphiregulin also differed between patients with and without CCM, but its discriminatory value was lower (AUROC 0.709), while PICP showed an AUROC of 0.662 [64]. Galectin-3 has also been associated with cardiac dysfunction and may have prognostic value, particularly when combined with BNP, but its specificity is limited because it is also increased in hepatic fibrosis and inflammation [47]. Thus, BNP, NT-proBNP and troponin may be useful as supportive markers, whereas amphiregulin, galectin-3 and other fibrosis-related markers remain investigational. Their interpretation should consider liver disease severity, volume status and renal function and should be integrated with echocardiographic and clinical findings [47,63].

Inflammatory and oxidative stress pathways, together with changes in vascular and nitric oxide regulation, may contribute to cardiac dysfunction in patients with cirrhosis [53,65]. Our previous review focused on oxidative stress and cirrhosis-associated cardiac dysfunction [65]. In the present review, the focus is on the diagnosis of CCM and its relationship with hypoxic hepatitis and congestive hepatopathy.

Markers of extracellular matrix remodeling and fibrosis, including hyaluronic acid, laminin, procollagen type III N-terminal peptide (PIIINP) and collagen type IV, have also been investigated, but their role in CCM remains uncertain [47,53]. Biomarkers are best interpreted as supportive findings alongside echocardiographic and clinical assessment, taking into account liver disease severity, volume status and renal function [47,63].

Current evidence indicates that biomarkers alone have limited diagnostic specificity for cirrhotic cardiomyopathy. A combined approach integrating natriuretic peptides with speckle-tracking echocardiography, diastolic function parameters, stress testing and functional capacity assessment may improve detection of CCM [46,49]. Future diagnostic strategies will likely rely on integrated cardiohepatic assessment combining multimodal imaging, biomarkers and functional testing to improve phenotypic characterization and individualized cardiovascular risk stratification in cirrhosis.

The clinical, laboratory, and imaging features of hypoxic hepatitis, congestive hepatopathy, and cirrhotic cardiomyopathy are summarized in Figure 1. A practical diagnostic approach to these conditions is presented in Figure 2.

### 5.5. Treatment and Clinical Significance of Cirrhotic Cardiomyopathy

There is currently no disease-specific therapy for CCM. Management primarily focuses on optimal treatment of the underlying liver disease and correction of the hemodynamic abnormalities associated with cirrhosis. Standard heart failure therapies may be considered in patients who develop overt systolic dysfunction; however, their use is frequently limited by systemic hypotension, renal dysfunction, and altered circulatory physiology. Liver transplantation remains the only intervention capable of reversing many of the functional and structural cardiac abnormalities associated with CCM, although recovery may be incomplete in advanced disease.

Acute decompensation, severe infection, gastrointestinal bleeding, hepatorenal syndrome, major surgery, TIPS (Transjugular intrahepatic portosystemic shunt) implantation or liver transplantation may all unmask previously subclinical cardiac dysfunction and precipitate overt heart failure, arrhythmias, pulmonary edema or circulatory instability. Accordingly, CCM should be regarded as a clinically relevant component of cirrhosis rather than merely an incidental echocardiographic finding [42,43,44]. Cardiovascular assessment before liver transplantation includes clinical evaluation, ECG, and transthoracic echocardiography [66]. In patients without signs of coronary heart disease, assessment using the CAD-LT (coronary artery disease in liver transplantation) score is recommended [66]. Based on the CAD-LT score, patients are stratified into low-, intermediate-, or high-risk groups, with further evaluation by coronary CT angiography or conventional coronary angiography, as appropriate [66]. Coronary angiography is most often indicated during pretransplant evaluation in patients with signs of coronary heart disease [66]. Pretransplant measurement of BNP and hs-troponin I may help in stratifying the risk of early post-transplant mortality [66]. Assessment of frailty and physical capacity is also necessary to predict perioperative mortality and morbidity [66].

TIPS implantation represents one of the most important clinical settings in which impaired cardiovascular reserve becomes evident. The abrupt increase in venous return and preload following TIPS may exceed the adaptive capacity of the cirrhotic heart, particularly in patients with latent systolic or diastolic dysfunction. Similarly, liver transplantation is associated with major perioperative hemodynamic and inflammatory stress that may reveal previously unrecognized myocardial dysfunction [7,44,48]. Contemporary cardiovascular assessment may therefore improve pretransplant cardiovascular risk stratification beyond conventional echocardiographic assessment alone [42,48].

Isolated heart transplantation (HT) is considered an appropriate treatment option in patients with advanced heart failure who have no significant liver disease or who have non-cirrhotic liver injury with a normal hepatic venous pressure gradient (HVPG ≤ 5 mmHg) [67]. In these patients, hepatic dysfunction, particularly when related to cardiac congestion, may improve after restoration of cardiac function. Advanced liver disease and cirrhosis are considered contraindications to isolated HT [67], as irreversible hepatic injury is unlikely to improve after correction of the underlying cardiac disease. In a small series of patients with cirrhosis undergoing isolated HT, short-term mortality was 50% and major in-hospital complications occurred in 75% of patients [68]. These findings highlight the need for careful pre-transplant assessment of both cardiac and hepatic function and for a multidisciplinary approach. The evaluation may include liver imaging, assessment of hepatic synthetic function, and, when advanced liver disease cannot be reliably excluded, transjugular liver biopsy with measurement of the hepatic venous pressure gradient. When advanced and irreversible liver disease is present in a patient who also requires heart transplantation, combined heart–liver transplantation (CHLT) may be considered. Universally accepted criteria for CHLT have not yet been established [67]. Cardiac cirrhosis is among the most common indications for CHLT [67].

TIPS is an important treatment option for selected patients with complications of portal hypertension, particularly variceal bleeding and refractory ascites [69]. TIPS increases venous return and cardiac preload and may precipitate or worsen pre-existing cardiac dysfunction. Elective TIPS should therefore be avoided in patients with LVEF < 50% because of the risk of cardiac decompensation [69]. Severe pulmonary hypertension, particularly a mean pulmonary artery pressure > 45 mmHg, is also considered a contraindication to TIPS [69]. In patients with less severe cardiac dysfunction, TIPS may be considered after careful cardiac assessment and multidisciplinary evaluation, with the potential benefits of portal decompression weighed against the risk of cardiac decompensation [69]. Cardiovascular assessment before TIPS placement includes a detailed medical history, physical examination, ECG, and measurement of NT-proBNP [70]. If any of these assessments suggest an abnormality, echocardiography should be performed [70].

Importantly, cardiovascular abnormalities associated with CCM may also carry prognostic significance. Impaired global longitudinal strain, diastolic dysfunction, elevated natriuretic peptide levels, and reduced functional reserve have all been associated with more advanced liver disease and adverse clinical outcomes in selected studies [46,48,49]. Although available evidence remains heterogeneous, these observations suggest that early recognition of impaired cardiovascular reserve may improve risk stratification and multidisciplinary management in patients with advanced cirrhosis.

## 6. Conclusions

The heart and liver are closely interconnected through hemodynamic, neurohumoral, inflammatory, and metabolic mechanisms. Cardiac disorders may lead to hepatic injury through hypoxic and congestive mechanisms, while advanced liver disease may be associated with clinically relevant cardiac dysfunction, particularly cirrhotic cardiomyopathy. Because these conditions may have overlapping clinical and laboratory findings, their diagnosis often requires a combination of clinical assessment, laboratory tests, echocardiography, and other imaging modalities. Conventional clinical, laboratory, and echocardiographic assessment remain the main components of the diagnostic evaluation. Newer approaches, including myocardial deformation imaging, advanced cardiac imaging, and emerging biomarkers, may provide additional information, but their diagnostic value is not yet sufficiently validated for routine clinical use. Further studies are needed to establish more consistent diagnostic criteria and to better distinguish hypoxic liver injury from hepatic congestion and progressive fibrosis. As a narrative review, the present article is limited by the heterogeneity of the available literature and by differences in study populations and diagnostic methods, which make direct comparison of findings difficult.

## Figures and Tables

**Figure 1:**
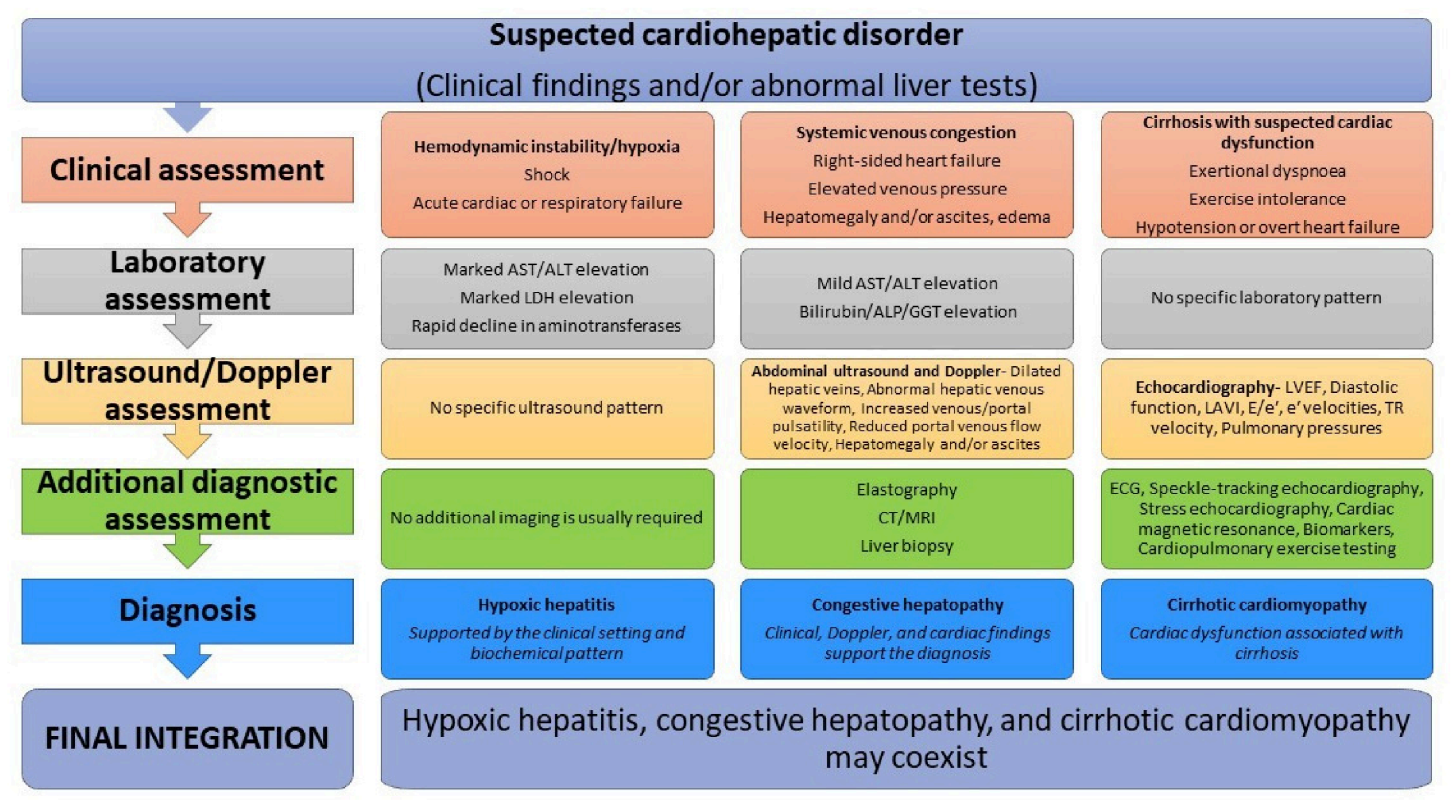
Comparative summary of clinical, laboratory, and imaging features of hypoxic hepatitis, congestive hepatopathy, and cirrhotic cardiomyopathy. AST—Aspartate aminotransferase; ALT—Alanine aminotransferase; LDH—Lactate dehydrogenase; GGT—Gamma-glutamyl transferase; LVEF—Left ventricular ejection fraction; LAVI—Left atrial volume index; E/e′—Ratio of early transmitral flow velocity (E) to early diastolic mitral annular velocity (e′); TR—Tricuspid regurgitation; CT—Computed tomography; MRI—Magnetic resonance imaging; ECG—Electrocardiogram.

**Figure 2:**
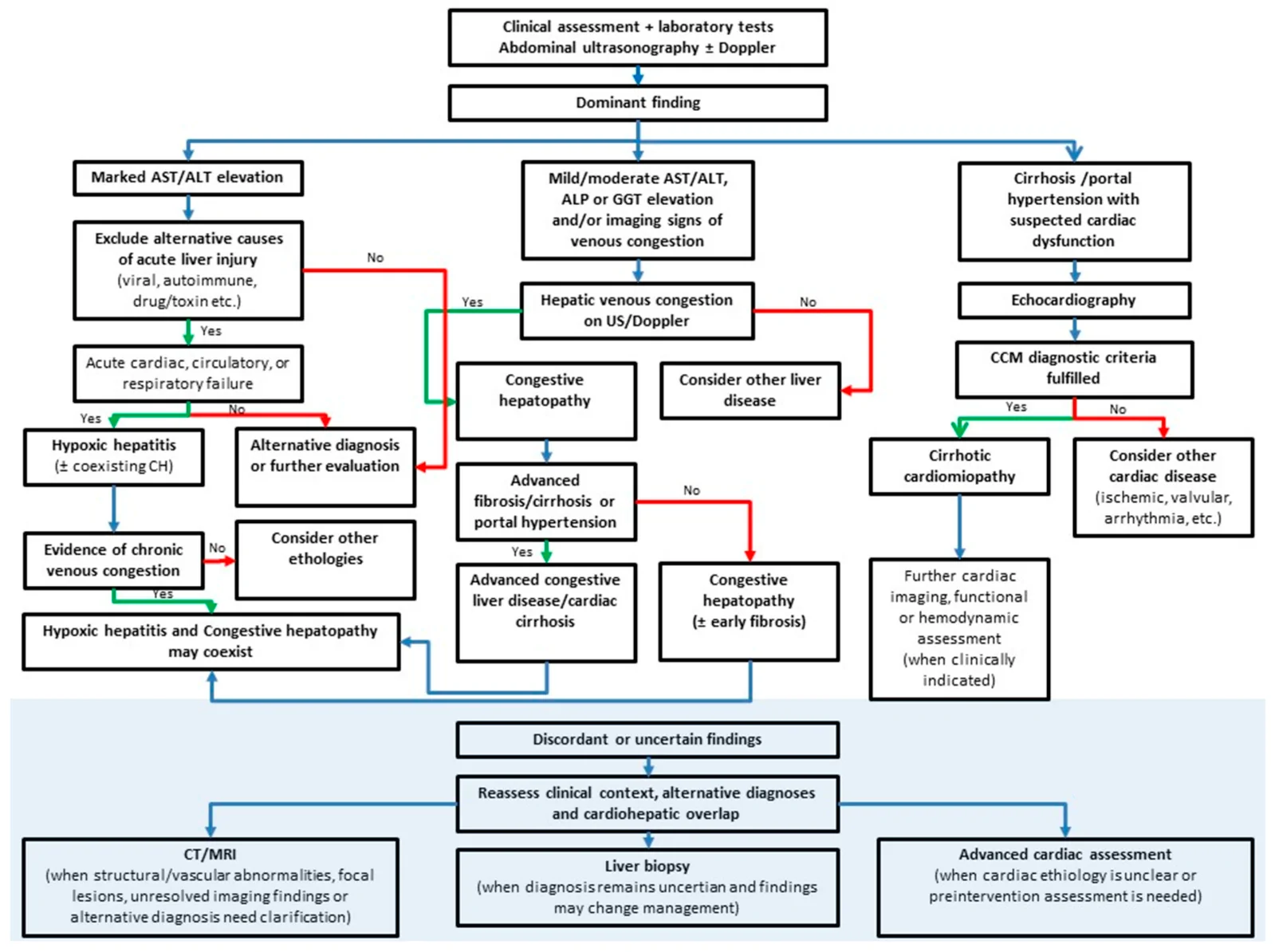
Practical diagnostic approach to hypoxic hepatitis, congestive hepatopathy, and cirrhotic cardiomyopathy. AST—Aspartate aminotransferase; ALT—Alanine aminotransferase; GGT—Gamma-glutamyl transferase; CH—Congestive hepatopathy; CCM—Cirrhotic cardiomyopathy.

**Table 1:** Causes of Marked Aminotransferase Elevation Relevant to the Differential Diagnosis of Hypoxic Hepatitis.

Etiology	Key Distinguishing Laboratory Value	Key Distinguishing Clinical Features
**Drug- and toxin-induced liver injury (DILI)**	Marked and variable aminotransferase elevation; bilirubin variable; cholestatic parameters depend on pattern of injury	Recent exposure to potentially hepatotoxic drug, supplement or toxin; temporal relationship between exposure and liver injury
**Autoimmune**	Marked aminotransferase elevation; increased IgG; bilirubin variable; autoimmune antibody	Acute or subacute presentation; autoimmune features may be present; positive autoantibodies; compatible liver histology; female predominance
**Infectious (viral)**	Marked aminotransferase elevation; ALT often predominant; LDH usually less prominent than in hypoxic hepatitis; bilirubin may be substantially elevated; viral serology positivity	Prodromal symptoms may occur; exposure history; absence of primary hemodynamic insult;
**Vascular (Budd–Chiari syndrome, sinusoidal obstruction syndrome)**	Aminotransferases may be markedly elevated, particularly in acute vascular obstruction; bilirubin variable; abnormalities in coagulation may occur with severe hepatic dysfunction	Abdominal pain, hepatomegaly and/or ascites; risk factors for hepatic venous outflow obstruction or sinusoidal injury; vascular imaging demonstrates the underlying lesion
**Pregnancy-related (Acute fatty liver of pregnancy, HELLP syndrome)**	Aminotransferase elevation, usually accompanied by hyperbilirubinemia and/or other abnormalities reflecting hepatic dysfunction; HELLP: hemolysis, elevated liver enzymes and low platelet count; acute fatty liver of pregnancy may be associated with hypoglycemia and coagulopathy	Usually occurs in late pregnancy; nausea, vomiting, abdominal pain or malaise may occur; hypertension/proteinuria and thrombocytopenia support HELLP; clinical features of acute hepatic dysfunction may occur in acute fatty liver of pregnancy
**Biliary disorders**	Aminotransferases may be markedly elevated during acute biliary obstruction, sometimes preceding a predominantly cholestatic pattern; bilirubin and alkaline phosphatase may subsequently increase	Acute right upper quadrant/epigastric pain, jaundice and/or fever may occur; imaging may demonstrate biliary obstruction, gallstones or ductal dilatation
**Metabolic**	Usually mild-to-moderate aminotransferase elevation rather than a sustained marked elevation; pattern depends on the underlying metabolic disorder; hemolysis in Wilson disease	Metabolic risk factors and/or features of metabolic dysfunction; marked acute elevations should prompt evaluation for alternative acute hepatocellular causes
**Genetic**	Laboratory abnormalities depend on the specific inherited disorder; Wilson disease may present with marked aminotransferase elevation and acute liver failure, often with disproportionate bilirubin elevation and a low alkaline phosphatase relative to bilirubin	Young age, family history or extrahepatic manifestations may provide diagnostic clues; Wilson disease should be considered particularly in young patients with acute liver failure or unexplained acute hepatitis
**Infiltrative/Malignant**	Aminotransferase elevation is usually less prominent than alkaline phosphatase elevation; marked aminotransferase elevation is uncommon unless there is extensive or acute hepatic involvement	Weight loss, constitutional symptoms, hepatomegaly or known malignancy may be present; imaging may demonstrate focal or diffuse hepatic infiltration

**Table 2:** Differential Diagnosis of Hypoxic Hepatitis and Congestive Hepatopathy.

Characteristics	Hypoxic Hepatitis	Congestive Hepatopathy
**Onset**	Acute	Chronic
**Main pathophysiological mechanism**	Hepatic hypoperfusion and ischemia	Hepatic congestion
**Aminotransferase**	Markedly elevated (>20× upper limit of normal)	Mildly elevated (usually 2–3× upper limit of normal)
**Bilirubin**	Normal or mildly elevated	Mildly elevated (cholestatic pattern)
**ALP and GGT**	Normal or mildly elevated	Mildly elevated
**LDH**	Markedly elevated (>10–20× upper limit of normal)	Normal or mildly elevated
**Abdominal ultrasound**	Normal/nonspecific findings	Hepatomegaly, dilation of hepatic veins, ascites

Based on References [8,17,22]. ALP—alkaline phosphatase; GGT—gamma-glutamyl transferase; LDH—lactate dehydrogenase.

**Table 3:** Echocardiographic Parameters for the Assessment of Cirrhotic Cardiomyopathy.

Echocardiographic Domain	Parameter	Reference/Commonly Used Threshold *	Clinical Relevance in CCM (Role in Assessment)
**Left ventricular systolic function**	LVEF	≤50%	Overt systolic dysfunction; often normal at rest in early CCM (Formal criterion)
	LV GLS	Absolute GLS < 18%	Earliest marker of subclinical LV systolic dysfunction (Formal criterion)
**Left ventricular diastolic function**	Septal e′	<7 cm/s	Impaired myocardial relaxation (Formal criterion **)
	E/e′ ratio	≥15	Elevated LV filling pressures (Formal criterion **)
	LAVI	>34 mL/m^2^	Chronic diastolic dysfunction (Formal criterion **)
	Peak TR velocity	>2.8 m/s	Increased pulmonary pressure; integrated diastolic assessment (Formal criterion **—interpret cautiously in pulmonary or portopulmonary hypertension)
**Right ventricular function**	TAPSE	<17 mm	Reduced longitudinal RV systolic function (Supportive/complementary)
	RV S′	<9.5 cm/s	Impaired RV contractility (Supportive/complementary)
	RV FAC	<35%	Global RV systolic dysfunction (Supportive/complementary)
	RV free-wall strain	Less negative than −20%	Early RV dysfunction, often before TAPSE declines (Emerging/investigational)
	TAPSE/PASP	<0.55 mm/mmHg	RV–pulmonary artery uncoupling; prognostic marker (Prognostic/complementary)
**Right heart remodeling**	RA area	>18 cm^2^	Chronic venous congestion
	RV basal diameter	>41 mm	RV dilatation
**Pulmonary circulation**	PASP	Elevated according to current recommendations	Increased RV afterload; evaluate for portopulmonary hypertension (Supportive/complementary)
**SECHO**	Contractile reserve	Absent/inadequate increase in EF or GLS	Unmasks latent systolic dysfunction characteristic of CCM

Based on References [7,42,47]. * The formal diagnostic criteria for CCM include LVEF and absolute GLS for systolic dysfunction, and the presence of at least three of four specified echocardiographic abnormalities for advanced diastolic dysfunction. Other parameters are supportive, complementary, prognostic, or investigational and should not be interpreted as independent diagnostic criteria. ** Advanced diastolic dysfunction is defined by the presence of at least three of these four echocardiographic abnormalities. FAC—fractional area change; GLS—global longitudinal strain; LAVI—left atrial volume index; PASP—pulmonary artery systolic pressure; RA—right atrium; RV—right ventricle; TAPSE—tricuspid annular plane systolic excursion; SECHO—stress echocardiography.

## Data Availability

No new data were created or analyzed in this study. Data sharing is not applicable to this article.

## Supplementary Materials

The following supporting information can be downloaded at: https://www.mdpi.com/article/10.3390/diagnostics16193126/s1, Table S1. Complete literature search strategies used in PubMed and Web of Science Core Collection.
