# Supplementary material for: Heart–Liver Interactions: Pathophysiology and Diagnosis of Hypoxic Hepatitis, Congestive Hepatopathy, and Cirrhotic Cardiomyopathy
Source: Diagnostics (Basel). 2026 Sep 26;16(19):3126. doi: 10.3390/diagnostics16193126 (PMC13664619; doi:10.3390/diagnostics16193126)
Supplement: Supplementary file 1 [file diagnostics-16-03126-s001.zip › diagnostics-4517498-supplementary.pdf]

Supplementary Table S1. Complete literature search strategies used in PubMed and Web of Science Core Collection

Database	Search domain	Complete search strategy	Date range	Language	Records retrieved
**PubMed**	Ischemic/hypoxic hepatitis	("ischemic hepatitis"[Title/Abstract] OR "ischaemic hepatitis"[Title/Abstract] OR "hypoxic hepatitis"[Title/Abstract] OR "hypoxic liver injury"[Title/Abstract] OR "ischemic liver injury"[Title/Abstract] OR "ischaemic liver injury"[Title/Abstract] OR "shock liver"[Title/Abstract]) AND ("2000/01/01"[Date - Publication] : "2026/06/30"[Date - Publication]) AND English[Language]	01 Jan 2000–30 Jun 2026	English	**155**
**PubMed**	Congestive hepatopathy	("congestive hepatopathy"[Title/Abstract] OR "congestive liver disease"[Title/	01 Jan 2000–30 Jun 2026	English	**350**

Database	Search domain	Complete search strategy	Date range	Language	Records retrieved
		Abstract] OR "cardiac hepatopathy"[Title/Abstract] OR "cardiac liver disease"[Title/Abstract] OR "cardiac cirrhosis"[Title/Abstract] OR "cardiac-induced liver disease"[Title/Abstract] OR "heart failure-associated liver disease"[Title/Abstract] OR "heart failure associated liver disease"[Title/Abstract]) AND ("2000/01/01"[Date - Publication] : "2026/06/30"[Date - Publication]) AND English[Language]			
**PubMed**	Cirrhotic cardiomyopathy	("cirrhotic cardiomyopathy"[Title/Abstract] OR "cirrhosis-associated cardiomyopathy"[Title/Abstract] OR	01 Jan 2000–30 Jun 2026	English	**509**

Database	Search domain	Complete search strategy	Date range	Language	Records retrieved
		"cirrhosis associated cardiomyopathy"[Title/Abstract] OR "cirrhosis-related cardiomyopathy"[Title/Abstract] OR "cirrhosis related cardiomyopathy"[Title/Abstract] OR "cirrhotic cardiac dysfunction"[Title/Abstract] OR "cirrhosis-associated cardiac dysfunction"[Title/Abstract] OR "cirrhosis associated cardiac dysfunction"[Title/Abstract]) AND ("2000/01/01"[Date - Publication] : "2026/06/30"[Date - Publication]) AND English[Language]			
**PubMed**	Heart–liver axis/cardiohepatic syndromes	("heart-liver axis"[Title/Abstract] OR "heart liver axis"[Title/Ab	01 Jan 2000–30 Jun 2026	English	**26**

Database	Search domain	Complete search strategy	Date range	Language	Records retrieved
		stract] OR "cardiohepatic syndrome"[Title/Abstract] OR "cardio-hepatic syndrome"[Title/Abstract] OR "hepato-cardiac syndrome"[Title/Abstract] OR "cardiac-hepatic syndrome"[Title/Abstract] OR "heart-liver interaction"[Title/Abstract] OR "heart liver interaction"[Title/Abstract]) AND (diagnosis[Title/Abstract] OR diagnostic[Title/Abstract] OR pathophysiolog*[Title/Abstract] OR biomarker*[Title/Abstract] OR imaging[Title/Abstract] OR echocardiograph*[Title/Abstract] OR ultrasonograp			

Database	Search domain	Complete search strategy	Date range	Language	Records retrieved
		h*[Title/Abstract] OR elastograph*[Title/Abstract] OR "magnetic resonance"[Title/Abstract] OR CMR[Title/Abstract]) AND ("2000/01/01"[Date - Publication] : "2026/06/30"[Date - Publication]) AND English[Language]			
**Web of Science Core Collection**	Ischemic/hypoxic hepatitis	TS=("ischemic hepatitis" OR "ischaemic hepatitis" OR "hypoxic hepatitis" OR "hypoxic liver injury" OR "ischemic liver injury" OR "ischaemic liver injury" OR "shock liver") AND DOP=2000-01-01/2026-06-30	01 Jan 2000–30 Jun 2026	English	**648**
**Web of Science Core Collection**	Congestive hepatopathy	TS=("congestive hepatopathy" OR "congestive liver disease" OR "cardiac hepatopathy"	01 Jan 2000–30 Jun 2026	English	**456**

Database	Search domain	Complete search strategy	Date range	Language	Records retrieved
		OR "cardiac liver disease" OR "cardiac cirrhosis" OR "cardiac-induced liver disease" OR "heart failure-associated liver disease" OR "heart failure associated liver disease") AND DOP=2000-01-01/2026-06-30			
**Web of Science Core Collection**	Cirrhotic cardiomyopathy	TS=("cirrhotic cardiomyopathy" OR "cirrhosis-associated cardiomyopathy" OR "cirrhosis associated cardiomyopathy" OR "cirrhosis-related cardiomyopathy" OR "cirrhosis related cardiomyopathy" OR "cirrhotic cardiac dysfunction" OR "cirrhosis-associated cardiac dysfunction"	01 Jan 2000–30 Jun 2026	English	**781**

Database	Search domain	Complete search strategy	Date range	Language	Records retrieved
		OR "cirrhosis associated cardiac dysfunction") AND DOP=2000-01-01/2026-06-30			
**Web of Science Core Collection**	Heart–liver axis/cardiohepatic syndromes	TS=("heart-liver axis" OR "heart liver axis" OR "cardiohepatic syndrome" OR "cardio-hepatic syndrome" OR "hepato-cardiac syndrome" OR "cardiac-hepatic syndrome" OR "heart-liver interaction" OR "heart liver interaction") AND TS=(diagnosis OR diagnostic OR pathophysiolog* OR biomarker* OR imaging OR echocardiograph* OR ultrasonograph* OR elastograph* OR "magnetic	01 Jan 2000–30 Jun 2026	English	**29**

Database	Search domain	Complete search strategy	Date range	Language	Records retrieved
		resonance" OR CMR) AND DOP=2000-01-01/2026-06-30			

Abbreviations: CMR, cardiac magnetic resonance; DOP, Date of Publication; TS, Topic field in Web of Science.
